# The complete chloroplast genome sequence of *Catalpa fargesii* f. *duclouxii* (Bignoniaceae)

**DOI:** 10.1080/23802359.2020.1823267

**Published:** 2020-09-29

**Authors:** Wen-Jun Ma, Song-Zhi Xu, Gui-Juan Yang, Ying Liu, Xiao-Guo Xiang, Jun-Hui Wang

**Affiliations:** a State Key Laboratory of Tree Genetics and Breeding, Key Laboratory of Tree Breeding and Cultivation of Forestry and Grassland Administration, Research Institute of Forestry, Chinese Academy of Forestry, Beijing, China; b School of Life Science, Nantong University, Nantong, China; c Jiangxi Province Key Laboratory of Watershed Ecosystem Change and Biodiversity, Institute of Life Science and School of Life Sciences, Nanchang University, Nanchang, China

**Keywords:** Bignoniaceae, *Catalpa fargesii* f. *duclouxii*, phylogeny, chloroplast genome

## Abstract

The complete chloroplast genome sequence of *Catalpa fargesii* f. *duclouxii* C.A. May was firstly assembled and analyzed in this study. The whole genome of this species was 158164 bp in length, with a typical quadripartite structure. The large single copy (LSC) was 83986 bp, the small single copy (SSC) was 12660 bp, and both of the two inverted repeats (IRs) were 30259 bp, respectively. A total of 134 genes in the chloroplast genome were annotated, including 87 protein-coding genes, 8 ribosomal RNA (rRNA) genes, and 39 transfer RNA (tRNA) genes. The phylogenetic analysis showed that *C. fargesii* f. *duclouxii* was highly clustered with *C. bungei*.

*Catalpa fasrgesii f*. *duclouxii* (Bignoniaceae) is deciduous tree that endemic to southwestern China. It is a famous ornamental tree species for having showy flowers and produces valuable timber for furniture, boatbuilding, instrument (Li 2013). Besides, the whole plant of *C. fargesii* f. *duclouxii* can be used as medicine for the treatment of stomach, cough, rheumatalgia.

*Catalpa fargesii* f*. duclouxii* was collected from the type locality Guiding county, Guizhou province, China (26°41′45.28″N, 107°14′26.94″E). The voucher specimen (Ma WJ, DQ-1, CAF!) was deposited at the herbarium of Research Institute of Forestry, Chinese Academy of Forestry, Beijing, China. Total genomic DNA was extracted from silica gel-dried leaves using the modified CTAB procedure of Doyle and Doyle (1987). For short-read sequencing, an ∼150 bp insert size pair-end library was constructed and sequenced using the Illumina HiSeq 2000 platform in the Novogene Bioinformatics Institute (Beijing, China). A total of 5.6 Gb raw data were generated. By using SPAdes 3.13.0 (Bankevich et al. 2012) and Geneious 9.0.5 (http://www.geneious.com/), all contigs of the chloroplast genome sequence were spliced and assembled. Then, the webserver DOGMA (Wyman et al. 2004) was applied to annotate the complete chloroplast genome and Simple sequence repeats (SSR) were detected by MISA (http://pgrc.ipk-gatersleben.de/misa). The whole chloroplast genome of *C. ovata* (MT186670) was used as reference. The complete chloroplast genome sequence has been deposited in GenBank with an accession number MT783420.

The complete chloroplast genome of *C. fargesii* f*. duclouxii* is 1,58,164 bp in length, inclusive of a typical quadripartite structure with two inverted repeats (IRs) of 30,259 bp separated by a large single copy (LSC) of 83,986 bp and a small single copy (SSC) of 12,660 bp. A total of 134 genes in the chloroplast genome, including 87 protein-coding genes, 8 ribosomal RNA (rRNA) genes and 39 transfer RNA (tRNA) genes, were identified. Ten protein-coding genes, such as *rpl2, nadB, ndhA, rpoC1, atpF* contained one intron, respectively. Just *ycf3* contained two introns. Protein-coding regions (CDS) contribute 48.4% of the chloroplast genome and the total of GC content was 38.1%. In a word, the chloroplast genome of *C. fargesii* f*. duclouxii* showed a highly conserved genome structure of Bignoniaceae (Ma et al. 2020). Furthermore, 40 SSR sites are detected in the cp genome of *C. fargesii* f*. duclouxii*.

To determine the phylogenetic status of *C. fargesii* f*. duclouxii*, additional 25 complete chloroplast genomes of Bignoniaceae, together with 2 species as outgroup (Figure 1), were downloaded from NCBI. By using RAxML 8.2.8 (Stamatakis 2014) and MrBayes 3.2.6 (Ronquist and Huelsenbeck 2003), the phylogenetic analysis of Bignoniaceae was performed with GTR + I+G model as determined by the Akaike information criterion (AIC) in Modeltest 3.7 (Posada and Crandall 1998). For BI analysis, four chains of the Markov Chain Monte Carlo (MCMC) were run, sampling one tree every 1000 generations for 5,000,000 starting with a random tree. Majority-rule (>50%) consensus trees were constructed after removing the burn-in period samples (the first 20% of the sampled trees). For ML analyses, we conducted a rapid bootstrap analysis (1000 replicates) and searched for the best-scoring ML tree simultaneously. Results showed that *C. fargesii* f*. duclouxii* was strongly supported as a sister to *C. bungei* (ML-BS = 100, BI-PP = 1.00). The phylogenetic relationship of Bignoniaceae here is congruent with that of Olmstead et al. (2009).

**Figrue 1.**
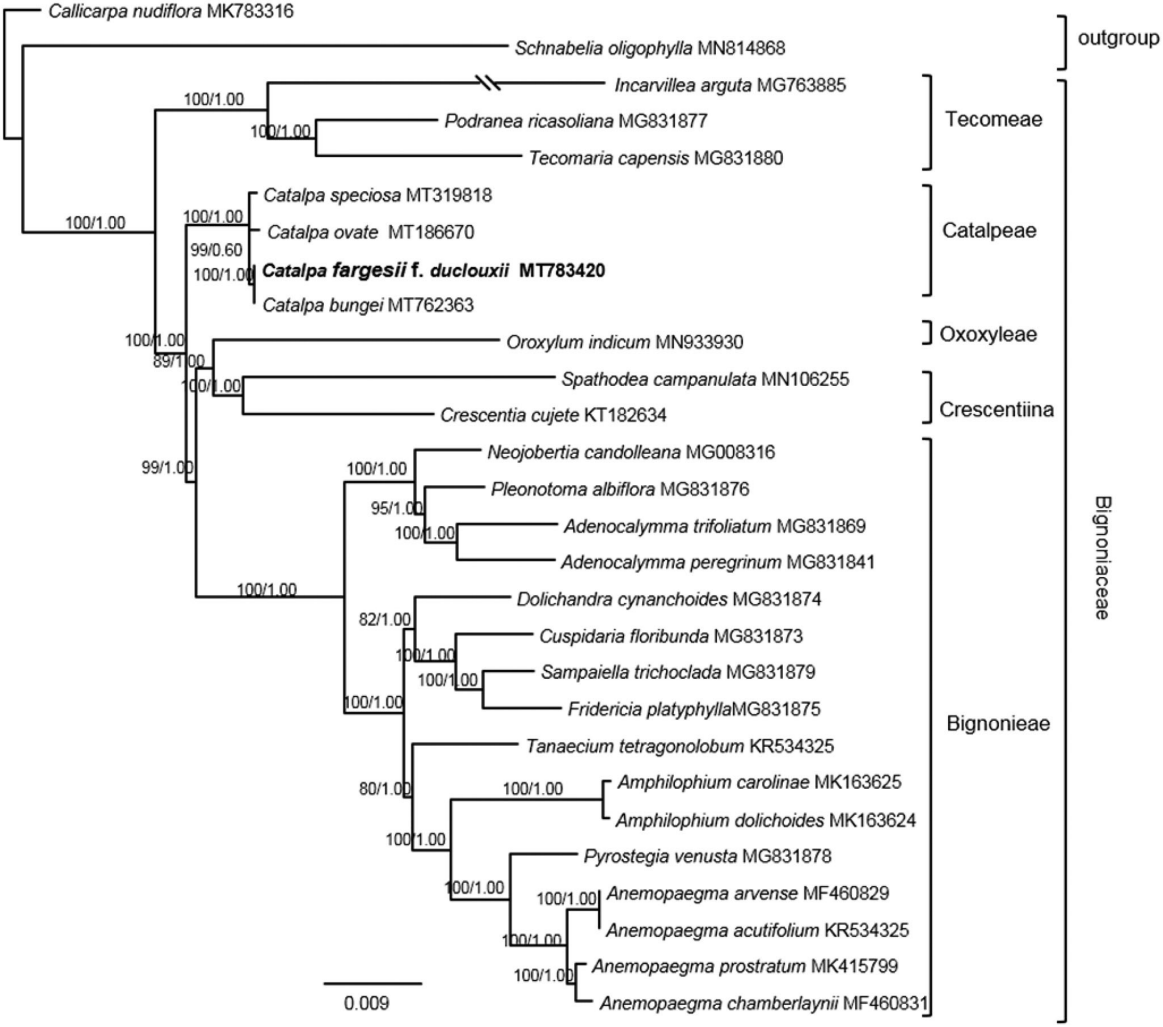
Maximum likelihood phylogram Bignoniaceae inferred from 27 chloroplast genomes. Numbers above branches indicated the maximum likelihood bootstrap support and the posterior probabilities, respectively.

## Data Availability

The data that support the finding of this study is available in GenBank at https://www.ncbi.nlm.nih.gov/ with an accession number MT783420.
